# Deep learning for semi-automated unidirectional measurement of lung tumor size in CT

**DOI:** 10.1186/s40644-021-00413-7

**Published:** 2021-06-23

**Authors:** MinJae Woo, A. Michael Devane, Steven C. Lowe, Ervin L Lowther, Ronald W. Gimbel

**Affiliations:** 1 Department of Public Health Sciences, Clemson University, 501 Edwards Hall, Clemson, SC 29634 USA; 2 Department of Radiology, Prisma Health System, 200 Patewood Drive, Greenville, SC 29615 USA

**Keywords:** Lung Cancer, Response Evaluation Criteria in Solid Tumors 1.1, Semi-automated annotation, Tumor Measurement, Deep learning

## Abstract

**Background:**

Performing Response Evaluation Criteria in Solid Tumor (RECISTS) measurement is a non-trivial task requiring much expertise and time. A deep learning-based algorithm has the potential to assist with rapid and consistent lesion measurement.

**Purpose:**

The aim of this study is to develop and evaluate deep learning (DL) algorithm for semi-automated unidirectional CT measurement of lung lesions.

**Methods:**

This retrospective study included 1617 lung CT images from 8 publicly open datasets. A convolutional neural network was trained using 1373 training and validation images annotated by two radiologists. Performance of the DL algorithm was evaluated 244 test images annotated by one radiologist. DL algorithm’s measurement consistency with human radiologist was evaluated using Intraclass Correlation Coefficient (ICC) and Bland-Altman plotting. Bonferroni’s method was used to analyze difference in their diagnostic behavior, attributed by tumor characteristics. Statistical significance was set at *p* < 0.05.

**Results:**

The DL algorithm yielded ICC score of 0.959 with human radiologist. Bland-Altman plotting suggested 240 (98.4 %) measurements realized within the upper and lower limits of agreement (LOA). Some measurements outside the LOA revealed difference in clinical reasoning between DL algorithm and human radiologist. Overall, the algorithm marginally overestimated the size of lesion by 2.97 % compared to human radiologists. Further investigation indicated tumor characteristics may be associated with the DL algorithm’s diagnostic behavior of over or underestimating the lesion size compared to human radiologist.

**Conclusions:**

The DL algorithm for unidirectional measurement of lung tumor size demonstrated excellent agreement with human radiologist.

**Supplementary Information:**

The online version contains supplementary material available at 10.1186/s40644-021-00413-7.

## Background

Response evaluation of cancer therapeutics is often a prerequisite to various clinical decisions in cancer treatment. Response Evaluation Criteria in Solid Tumor 1.1 (RECIST 1.1) is the predominant clinical guideline to determine whether tumors in cancer patients responded to treatment, stay the same, or worsened during cancer therapeutics [1–4]. Application of RECIST guideline involves a series of tumor size measurements, which is an important surrogate marker of therapeutic efficacy. Consistent and accurate measurements of tumor size are essential with their direct impact on cancer treatment management.

**Fig. 1:**
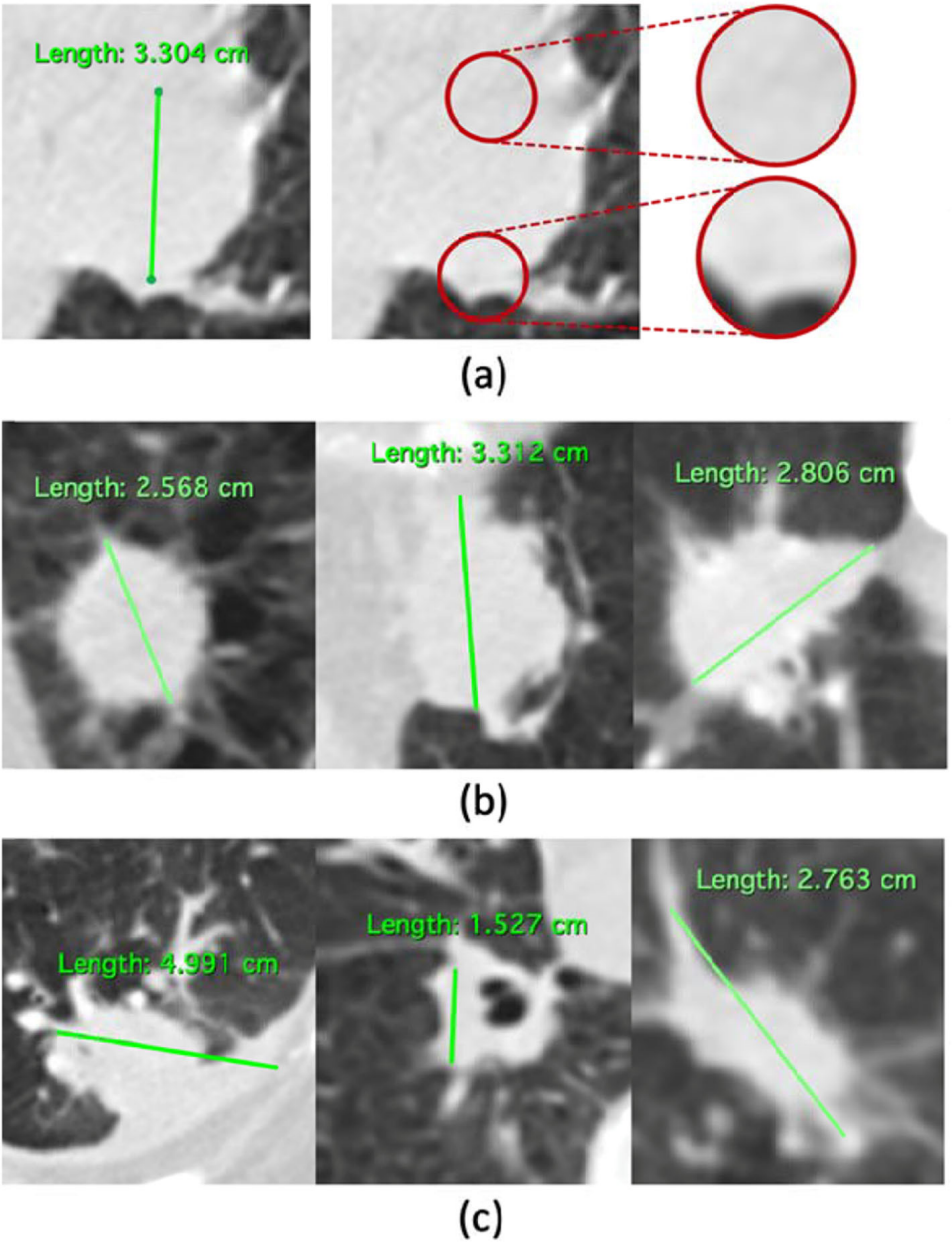
Challenges associated with lesion segmentation and its conversion to unidirectional RECIST measurement. **a** Automated lesion measurement is challenged by the absence of visual distinction between beginning and ending points and their surrounding areas. **b** Lesion boundaries are visually distinct and well-defined; both segmentation and conversion of segmentation into measurement can easily be automated using existing techniques and tools. **c** Both segmentation and conversion of segmentation into measurement require a significant amount of clinical reasoning, which poses challenges to the idea of automated measurement through segmentation

Performing RECISTS measurement is a non-trivial task requiring a great deal of expertise and time by a highly trained radiologist. Multiple reports have indicated that the tumor size measurements using computed tomography (CT) scans are subjected to intra- and inter-observer variability with various environmental factors causing the variability [5–12]. To address these challenges, researchers have attempted to develop systems to assist with consistent lesion measurement through automated lesion segmentation or masking for CT images [13–18]. Most studies used segmentation techniques with probabilistic approaches to drawing lesion boundaries. However, segmentation results are often non-comparable to radiologist measurements as radiologists use unidirectional measurement. Conversion of segmentation results into unidirectional measurement poses challenges as the task requires additional clinical reasoning to decide the start point, end point, and longest axis of the measurement, Fig. 1. Performing segmentation often takes longer than performing unidirectional measurement by human radiologists; this incurs additional costs on the acquisition of training data for any automated system for measurement.

In this study, we propose a new approach for application of a deep learning (DL) algorithm on semi-automated CT measurement of lung lesions. To the best of our knowledge, this study was the first to propose semi-automated measurement of tumor without involving segmentation or masking process. The purpose is to develop a tool performing measurement comparable to radiologist measurement, which has a potential to assist radiologists with consistent RECIST annotation by improving inter-observer measurement variability. We also investigated how different lesion types challenge the proposed application of the DL algorithm.

## Methods

### Image data sets

We reviewed 8 publicly open datasets with 146,403 cross-sectional lung CT images from various institutions (Table 1) [19–27]. A total of 1,617 cross-sectional lung CT images were included in this study after applying the following inclusion criteria: (a) selected lesion should be measurable under RECIST 1.1 (b) selected image file contains complete Digital Imaging and Communications in Medicine (DICOM) pixel data with no corruption (c) lesion size should differ by 20 % when compared to the previously selected images if selected from the same patient (d) selected image has at least 5mm spacing to the previously selected images if selected from the same patient. Additionally, DICOM metadata relevant to image processing (e.g. pixel spacing, window/level settings) was inspected for all selected image files. In the selected images, CT scanning parameters were as follows: tube voltage of 100, 120, 130, and 140 kV, and tube current 30–543 mA, and slice thickness of 1.0–6.0 mm.

**Table 1:** Characteristics of Data Sets

Parameter	NSCLC-Radiomics	CPTAC-LUAD	LCTSC	QIN LUNG CT	TCGA-LUSC	SPIE-AAPM Lung CT Challenge	LungCT-Diagnosis	RIDER Lung CT
No. of Scans	844	8	60	47	74	70	61	64
No. of Images	52,072	1,676	9,593	3,954	36,518	22,489	4,682	15,419
No. of Annotated Images	955	8	96	114	67	139	119	119
Training Set	667	4	68	80	47	97	83	83
Age	66.5	69.8	68.1	63.9	71.4	62.7	NA	61.6
Male	446 (67)	3 (75)	30 (44)	6 (8)	33 (70)	33 (34)	0 (0)	0 (0)
Female	221 (33)	1 (25)	38 (56)	16 (20)	14 (30)	64 (66)	0 (0)	0 (0)
Gender – Not Known	0 (0)	0 (0)	0 (0)	58 (72)	0 (0)	0 (0)	83 (100)	83 (100)
Validation Set	144	2	14	17	10	21	18	18
Age	65.5	73	72.3	69.4	71.9	60.2	NA	66.1
Male	101 (70)	1 (50)	5 (36)	1 (6)	6 (60)	5 (24)	0 (0)	0 (0)
Female	43 (30)	1 (50)	9 (64)	7 (41)	4 (40)	16 (76)	0 (0)	0 (0)
Gender – Not Known	0 (0)	0 (0)	0 (0)	9 (53)	0 (0)	0 (0)	18 (100)	18 (100)
Test Set	144	2	14	17	10	21	18	18
Age	68.6	73	71.2	65.6	72.3	55.7	NA	63.2
Male	106 (74)	1 (50)	7 (50)	6 (35)	5 (50)	12 (57)	0 (0)	0 (0)
Female	38 (26)	1 (50)	7 (50)	0 (0)	5 (50)	9 (43)	0 (0)	0 (0)
Gender – Not Known	0 (0)	0 (0)	0 (0)	11 (65)	0 (0)	0 (0)	18 (100)	18 (100)
Characteristic								
Slice thickness (mm)	3.0	1.3—3.0	1.3—3.0	2.5—6.0	1.0—5.0	1.0	2.5—6.0	1.3
Increment (mm)	3.0	0.6—3.0	1.3—3.0	2.0—5.0	0.6—5.0	1.0	2.5—5.0	1.3
In-plane Resolution (mm)	0.97	0.56—1.37	0.98—1.37	0.59—0.95	0.55—0.98	0.55—0.90	0.59—0.95	0.51—0.90
Dataset Version	Version 2: Updated 2016/05/31	Version 4: Updated 2018/10/24	Version 1: Updated 2017/05/17	Version 2: Updated 2017/07/31	Version 3: Updated 2017/01/30	Version 2: Updated 2016/09/23	Version 1: Updated 2014/12/30	Version 2: Updated 2014/11/14

Note – Average age and gender ratio were calculated using available DICOM information for each of the selected images. Numbers indicate counts of subjects with percentages in parentheses, unless indicated otherwise

### Reference RECIST measurements

**Fig. 2:**
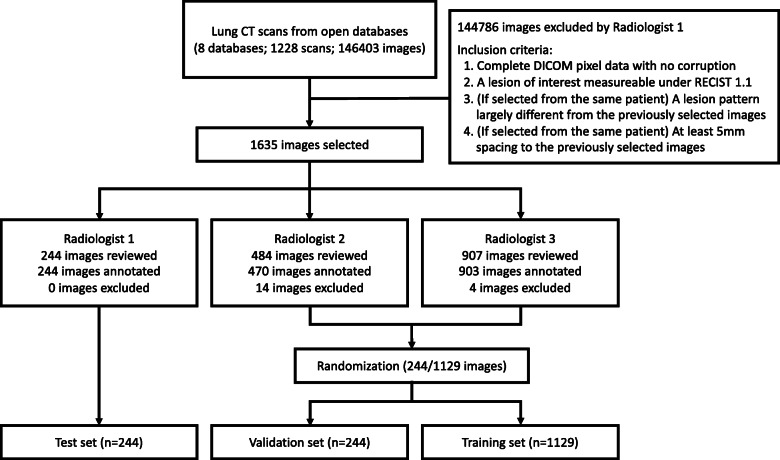
Flow diagram illustrating data collection procedures and inclusion criteria. The inclusion criteria were designed to ensure heterogeneity of lesion patterns in the collected data. Each radiologist independently performed the measurements. The participating radiologists were blind to the measurements performed by other radiologists

Three experienced board-certified radiologists who regularly perform treatment response evaluation participated in training and evaluation of the proposed DL algorithm, Fig. 2. Radiologist 1 (MD) selected CT images for the study according to the eligibility criteria. The 1,617 CT images selected by the Radiologist 1 were randomly assigned to training set, validation set, and test set through dataset-wise block randomization, Table 1. The following ratio was used as suggested in the potentially relevant studies using deep learning: 70 % training, 15 % validation, 15 % test [28–30]. Radiologist 2 (SL) and Radiologist 3 (EL) performed measurement on images from the training and validation sets, resulting in the exclusion of additional 18 images due to inter-observer variability regarding the RECIST measurability on the images initially selected by Radiologist 1. As a result, the training dataset included images deemed measurable by all three radiologists. The test set was labeled by Radiologist 1. For clarity, there was no overlap between training and test data. The radiologist who labeled the test dataset was ruled out during the training process. The participating radiologists did not have access to measurements performed by other radiologists to prevent observer bias. The reference measurements were performed between October 2018 to June 2019.

### Semi-automated measurement using deep learning algorithm

The DL network for automatic lesion measurement consisted of three consecutive convolutional neural networks that labeled whether the size of a target lesion in a given image frame was larger or smaller than 32 pixels. We assumed that, if the DL network failed to classify, the failure occurred because the lesion size approximated to 32 pixels.

The training data preparation was performed by resizing each CT image so that its target lesion would have a size of 32 pixels. The unidirectional measurements in centimeters were converted into measurements in pixels (${M}_{px}$). The images were then magnified by 32/${M}_{px}$ times using bicubic interpolation as a differentiable sampler for the different magnifications [31, 32]. Each target lesion was cropped in a 128-by-128 pixel frame using the center point of measurement as a frame center. The training dataset was generated through image augmentation techniques including zooming in/out, horizontal/vertical shifting of the target lesion in an image frame. Using the various magnifications, the DL network was trained to predict whether a lesion in a 128-by-128 pixel frame is larger or smaller than 32 pixels. The augmentation was also intended to improve the resilience of classification by training the DL algorithm with target lesions off the center of the image frame [33]. The convolutional neural network was trained for 500 iterations with a batch size of 32; the model with the highest validation accuracy was selected. The training process was not stratified by the lesion characteristics. A single DL classifier was utilized for both training and inference throughout the study.

**Fig. 3:**
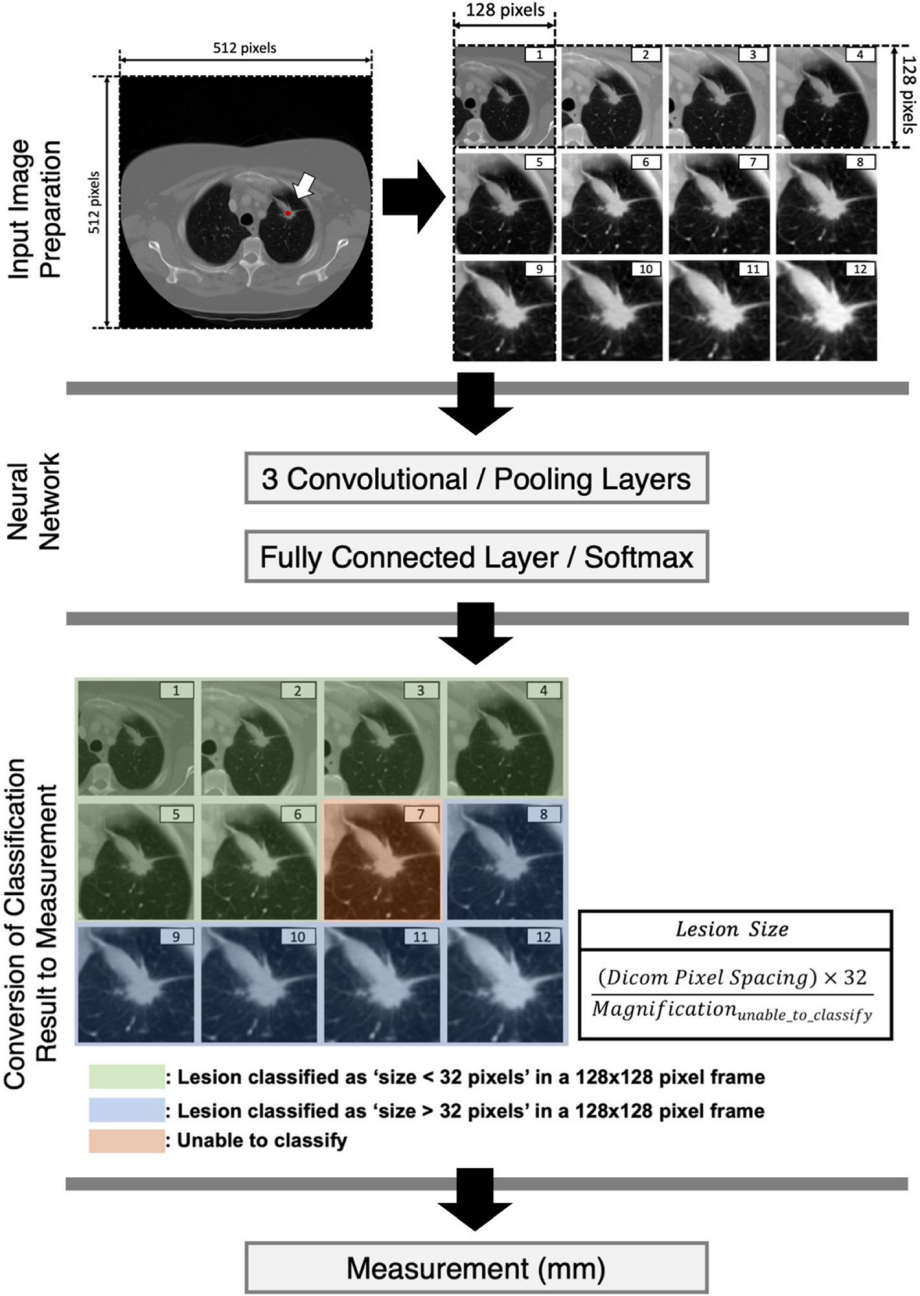
Overview of deep learning algorithm to perform unidirectional lesion measurement. In the shown example, the input image was augmented into 16 images at various magnifications, using the arbitrary input point (stained in red, pointed by arrow) in the target lesion. The neural network classified each augmented image whether the containing lesion size is larger or smaller than 32 pixels within a 128-by-128 pixel frame. Upon identification of a magnification inducing classification failure, the magnification and pixel spacing information were used to determine the final measurement

The proposed method was semi-automatic; the algorithm was first given with an arbitrary point within a target lesion to perform the RECIST measurements. Once the arbitrary point was acquired, the algorithm utilized the point as a frame center to cropped 128-by-128 pixel image frames containing target lesion with various magnifications, Fig. 3. Upon identification of magnification where the classification failed, a numerical value of measurement was calculated using the magnification and DICOM pixel spacing tag. The codes are available at https://github.com/minjaewoo/Semiautomated-CT-Measurement.

### Statistical analysis

Reliability of measurements by the DL algorithm was assessed with the intraclass correlation coefficient (ICC) between automatically and manually obtained measurements for images from the test set. The ICC was calculated using a two-way random-effects model that characterized absolute agreement to account for both lesion-wise effect (target effect) and radiologist-wise effect (rater effect) for evaluation comparable to the previous studies on inter-observer variability in CT measurement [11, 34].

**Fig. 4:**
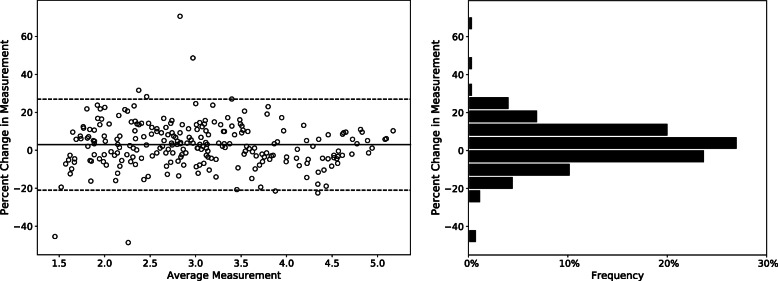
Bland-Altman plot and histogram illustrating measurement difference between human radiologist and DL algorithm. Each data point in Bland-Altman plot represent measurement difference between human radiologist and DL algorithm over the same lesion, calculated by the following formula: $\frac{2\bullet \left(measuremen{t}_{DL} - measuremen{t}_{human}\right)}{(measuremen{t}_{DL} + measuremen{t}_{human})}$. The limits of agreement are represented in the dotted line, calculated by the following formula: $\pm 1.96\bullet SD$. The horizontal solid lines represent systematic difference in Bland-Altman plot and histogram

Bland-Altman plotting with 95 % limits of agreement was produced by averaging lesion size between the human reader and DL algorithm to demonstrate the agreement between the measurements produced by the human reader and DL algorithm [35]. The percentage differences in measurement between the human and algorithm were visualized in a histogram, Fig. 4.

Additional statistical analyses were performed to identify the effect of lesion invasion type on variability between the human radiologist and the DL algorithm. Bonferroni’s method was used for pairwise comparison of measurement difference by type of tumor invasion. Statistical significance was set at *p* < 0.05.

## Results

### Characteristic of data sets

Mean ages of patients in the training, validation, and test sets were 66.9, 66.1, and 67.1, respectively, Table 1. Gender information extracted from DICOM metadata suggested more male (62 %) than female (38 %) representation in selected CT images. Average lesion sizes annotated by the human radiologists for training, validation, and test sets were 3.08 cm, 3.26 cm, and 2.99 cm, respectively, Table 2. As intended, Radiologist 1 annotated 244 images from the test set. Radiologist 2 and Radiologist 3 performed measurements on training and validation sets, which resulted in 903 and 470 images annotated by Radiologist 2 and Radiologist 3, respectively. The difference in the number of images annotated by each radiologist was mainly attributed to different measurement pace between the radiologists. The proposed data augmentation resulted in a total of 142,254 images for training. The augmented training data included 71,127 images with lesion size smaller than 32 pixels and 71,127 images with lesion size larger than 32 pixels within a 128-by-128 pixel frame.

**Table 2:** Reader statistics and inter-observer variability between human radiologist and DL algorithm

Reader	Image Use	Number of Annotated Images	Average Measurement (cm)
Radiologist 1	Test	244	2.99 ± 0.93 (1.57–4.91)
Radiologist 2	Training	734	3.17 ± 0.96 (1.51–5.00)
	Validation	159	3.21 ± 0.98 (1.50–4.99)
Radiologist 3	Training	395	2.92 ± 0.88 (1.49–4.94)
	Validation	85	3.35 ± 0.89 (1.56–4.79)
DL Algorithm	Test	244	3.07 ± 0.91 (1.37–5.44)
Radiologist 1 & DL Algorithm ICC: 0.959 (95% CI: 0.947, 0.967)			

Note – Average Measurement ± Standard Deviation. Numbers in parentheses represent range consisting of (minimum observed value – maximum observed value). ICC denotes intraclass correlation coefficient. The ICC score is based on a two-way random-effects model. CI denotes confidence interval.

### DL algorithm performance

**Fig. 5:**
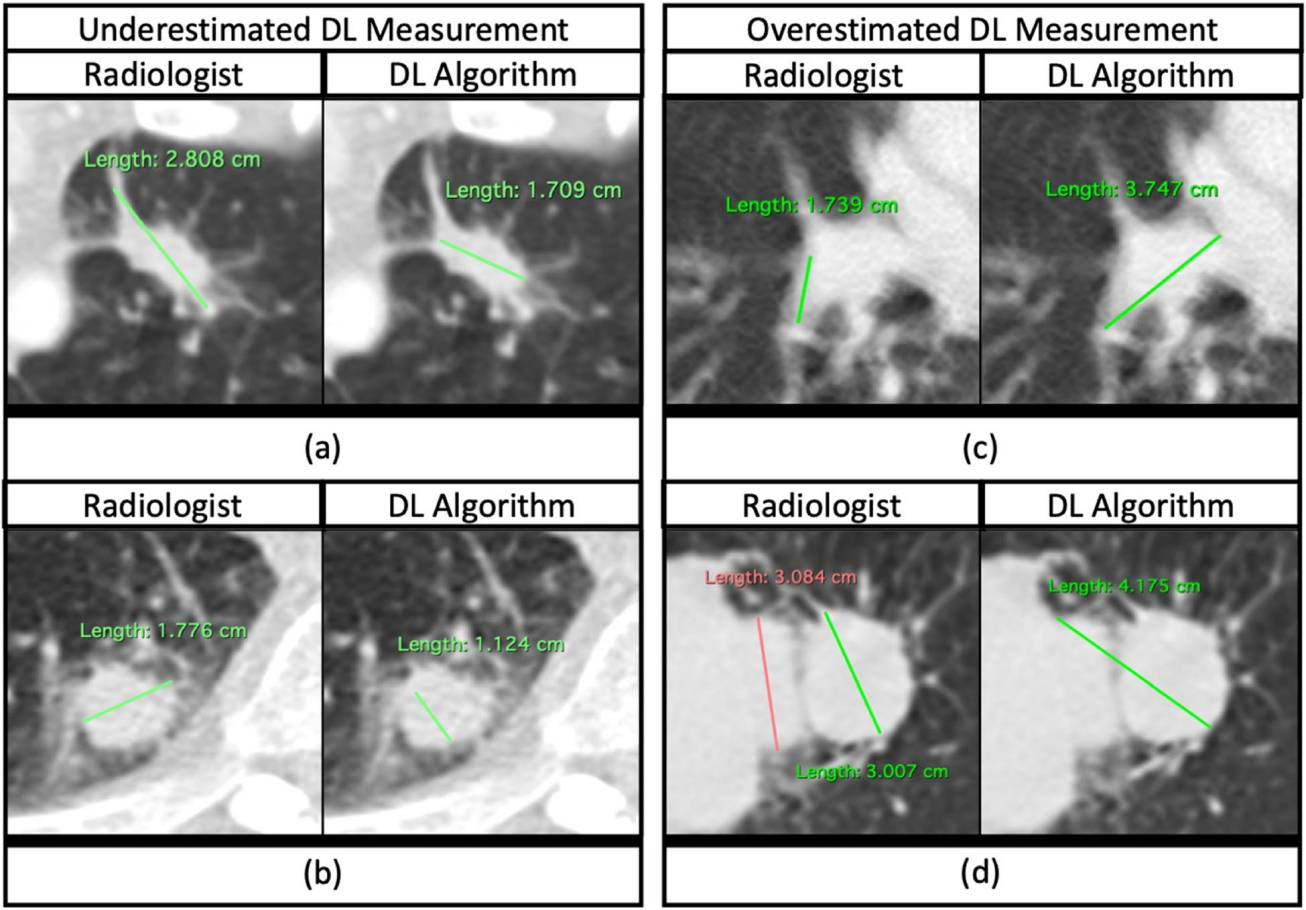
Example of outlier measurement differences from Bland-Altman plot. **a** The lesion underestimated by DL algorithm was subject to controversy on whether its spiculations should be included in the measurement or not. **b** The lesion was underestimated by DL algorithm with no clear clinical reasoning behind the measurement. **c** The lesion was overestimated by DL algorithm as the central density was included in the measurement **d** The lesion was overestimated by DL algorithm as the algorithm was interfered by two separate lesions sharing the same field of view

The DL algorithm achieved an ICC score of 0.959 (95 % CI: 0.947, 0.967) with Radiologist 1 when performing measurements on the same set of 244 CT images, Table 2. Bland-Altman plotting revealed a mean percent difference (systematic difference) of 2.97 % between human and DL algorithm; overall, the algorithm marginally overestimated the size of lesion by 2.97 % compared to human radiologists. Bland-Altman upper and lower limits of agreement (LOA) were realized at 24.3 and − 20.7 %. The plot also revealed a total of 6 measurements outside the lower and upper LOA. Although previous studies reported that the percent differences marginally outside the LOA are not unusual among human observers [12], some percent differences were unusually high and possibly indicated the algorithm failure. We have identified 4 lesions that caused unusually high measurement difference between the DL algorithm and human radiologist; there were two lesions above upper LOA with 48.5 and 70.6 % measurement difference, and two lesions below lower LOA with 45.4 and 48.6 % measurement difference. The lesions that caused the outlier measurement difference between human and DL algorithm were presented and compared in Fig. 5. For technical details on how the start point, end point, and longest axis were determined for the presented measurements by DL algorithm, see Supplemental Material 1.

The Bland-Altman analysis indicated no heteroscedasticity issue; the visualization suggested no evidence of increasing measurement difference between human and DL algorithm with an increase in average measurement. A benchmark to test the performance of the DL algorithm indicated that performing a single measurement by the algorithm takes on average of 2.2 s per lesion when tested on NVIDIA Jetson TX2 platform, whereas the participating radiologists spent on average of 17.8 s per lesion.

### Effect of invasion type on performance

**Fig. 6:**
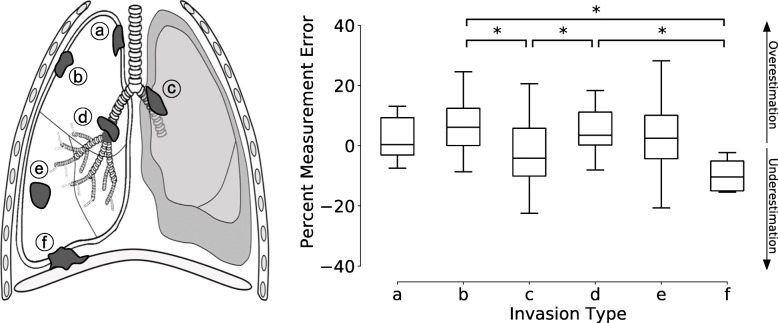
DL algorithm’s measurement error by tumor invasion type. The measurement error was calculated by using the following formula: $\frac{2\bullet \left(measuremen{t}_{DL} - measuremen{t}_{human}\right)}{(measuremen{t}_{DL} + measuremen{t}_{human})}$. The invasion types were classified as follows: **a** Parietal pleura/chest wall invasion. **b** Mediastinal pleural invasion. **c** Endobronchial invasion less than 2 cm distal to the carina. **d** Invasion associated with collapse (atelectasis). **e** Peripheral invasion surrounded by lung. **f** Diaphragm invasion. Box plots show the distribution of percent measurement errors stratified by invasion type. Bonferroni multiple comparison was performed, with statistical significance defined as *P < 0.05

Bland-Altman analysis suggested different diagnostic behavior between the DL algorithm and human radiologist when performing measurements. Overall, the algorithm tended to overestimate the size of lesion by 2.97 % compared to the human radiologist. Further comparisons stratified by tumor characteristics indicated that invasion type may be associated with diagnostic behavior of the DL algorithm resulting in overestimation or underestimation of lesion size. Specifically, a lesion-wise effect on the difference in diagnostic behavior between the human radiologist and DL algorithm was identified for the following invasion type classification: (A) Parietal pleura/chest wall invasion (B) Mediastinal pleural invasion (C) Endobronchial invasion less than 2 cm distal to the carina (D) Invasion associated collapse (atelectasis) (E) Peripheral invasion surrounded by lung (F) Diaphragm invasion, Fig. 6 [36]. The Bonferroni pairwise comparison suggested that the measurements by the algorithm are more likely to be overestimated compared to human radiologist when measuring (B) tumor invading mediastinal pleural and (D) tumor associated collapse (atelectasis) or obstructive pneumonia, as compared to when measuring (C) endobronchial tumor less than 2 cm distal to the carina and (F) tumor invading diaphragm.

## Discussion

This was the first study to perform semi-automated measurements without masking or segmentation process. The proposed algorithm facilitated the use of unidirectional measurement throughout its training process, which significantly reduced the cost of data acquisition. It yielded output comparable to a human radiologist’s standard RECIST measurement used in daily clinical practice. The proposed methodology has the potential to assist other anatomic measurements in the images with metadata containing information on pixel spacing.

The inter-observer agreement rate between the DL algorithm and human radiologist was 0.959 when evaluated using ICC. Its performance is consistent with the previously published study by McErlean in which 17 radiologists measured the same 320 lesions to evaluate inter-observer variability and achieved ICC scores of 0.943 and 0.967 among fellow and junior attending radiologists, respectively [11]. The proposed DL algorithm achieved an ICC score comparable to junior attending radiologists from the study when measuring the same set of 244 lesions. In a study by Tang et al., a convolutional neural network-based method for semi-automated RECISTS measurement was proposed and assessed using a mean difference between DL algorithm and radiologists in the unit of pixels (mean difference: 3.33 pixels; standard deviation: 4.93 pixels) [18]. Our model achieved a mean pixel difference and standard deviation of 2.85 and 2.51, which are 14 and 51 % lower than the performance suggested by the study, respectively. However, the score using pixel difference may not be a reliable measure as the score is largely affected by the composition of the dataset; when a percent measurement difference between readers is fixed, having a larger number of larger lesions may inflate the performance score of DL algorithm. For example, given a pixel spacing of 0.1 and a lesion size of 5 cm, the measurement difference of 5 pixels accounts only 10 % measurement difference between two readers. On the contrary, given a pixel spacing of 0.1 and a lesion size of 2 cm, the measurement difference of 5 pixels accounts 25 % measurement difference between two readers. In this study, we primarily used Bland-Altman plotting based on percent measurement difference to address the issue.

Bland-Altman plotting suggested that the proposed algorithm generally yielded comparable measurements to a human radiologist with 240 (98.4 %) measurements realized within or around the upper and lower limits of agreement (LOA). Among the 4 (1.6 %) measurements outside the LOA, we observed that 2 deviating measurements (Fig. 5a and c) potentially subject to controversy among human observers, with some radiologists accepting the measurements and others rejecting them. The first lesion underestimated by the DL algorithm (Fig. 5a) was subject to controversy on whether its spiculations should be included in the measurement or not. This particular case highlighted the inherent difficulty in measuring lung lesion as well as lesion in other organs, as there is no clear consensus existing with regard to how the spiculations should be taken into account in lesion measurement. In the second underestimated lesion (Fig. 5b), the clinical reasoning behind the underestimation by the DL algorithm is unclear. The first overestimated measurement by DL algorithm (Fig. 5c) appeared to include the central density in its measurement while the density was not included in the measurement by the human radiologist. The controversy was associated with whether the central density should be seen as a blood vessel or part of the target lesion. In the second overestimated lesion (Fig. 5d), the algorithm failed to recognize two separate lesions sharing the same field of view and combined them into a single measurement.

As demonstrated, the Bland-Altman plotting indicated that the algorithm tends to marginally overestimate the size of tumor compared to the human radiologist. Further statistical test was performed to assess whether the algorithm’s diagnostic behavior of under or overestimation is associated with the tumor types. It was observed that some tumor invasion types may induce the DL algorithm to over or underestimating the lesion size compared to the human radiologist. For example, the algorithm is likely to overestimate when measuring mediastinal pleural invasion (Fig. 6, Invasion Type b), compared to when measuring diaphragm invasion (Fig. 6, Invasion Type f) with the difference in its diagnostic behavior statistically significant. The lesion size measurement of some invasion types requires different clinical reasoning highly prone to inter-observer variability. The systematic difference between the algorithm and human radiologist attributed by the lesion-wise characteristics may or may not be due to inter-observer variability between the trainer and tester radiologists.

This study had some limitations. First, this was a semi-automated method as the algorithm requires an arbitrary point within target lesion as an input. Given the recent advancements in automated detection of lung cancer, future studies may want to address the limitation by exploring a hybrid model that detects a lesion, identifies an arbitrary point inside the lesion, and performs measurement using the input point. Second, it has been well documented that methods utilizing neural networks are subject to a well-recognized challenge of their black-box nature [37, 38], which make it harder to fully explain the algorithm’s measurement behavior. A further study utilizing techniques from interpretable machine learning may be explored to address the challenge [39, 40]. Lastly, the presented study was designed with an emphasis on internal validity by comparing the algorithm with one radiologist who was ruled out during the training process. Future study designs may aim at generalizability by training and comparing the algorithm with multiple groups of radiologists using external validation data set.

## Conclusions

This study proposed and validated a deep learning algorithm for semi-automated CT measurement of lung lesions. The DL algorithm yielded unidirectional measurements comparable to those of human radiologist and presented an excellent agreement. The DL algorithm was designed to work with any image with known patient-to-detector distance and the corresponding pixel spacing information, indicating a potential for its application in other anatomic measurements.

## Supplementary Information

**Additional file 1.**

## Data Availability

The datasets utilized during the current study are available in The Cancer Imaging Archive, https://www.cancerimagingarchive.net/.

## Abbreviations

ICC: Intraclass Correlation Coefficient
RECIST 1.1: Response Evaluation Criteria in Solid Tumors 1.1
DL: Deep Learning
DICOM: Digital Imaging and Communications in Medicine
LOA: Limits of agreement
