# Supplementary material for: Deep learning for semi-automated unidirectional measurement of lung tumor size in CT
Source: Cancer Imaging. 2021 Jun 23;21:43. doi: 10.1186/s40644-021-00413-7 (PMC8220702; doi:10.1186/s40644-021-00413-7)
Supplement: Supplementary file 1 — Additional file 1. [file 40644_2021_413_MOESM1_ESM.docx]

**Supplemental Material 1**

The presented DL algorithm offered only the numerical value of tumor size measurement. Additional image processing techniques were used to determine the following information for visualization purposes: (a) axis of measurement (b) either start or end point (i.e. vertex) of measurement.

**- Axis of Measurement**

The acquisition of the longest measurement axis was performed using image rotation and compression . The axis acquisition was based on idea that when the compression axis perfectly aligns with true measurement axis, there should be no change in the longest measurement value as a result of compression. Likewise, if the compression axis approximates to the true measurement axis, there should be minimal change in the longest measurement value after compression.


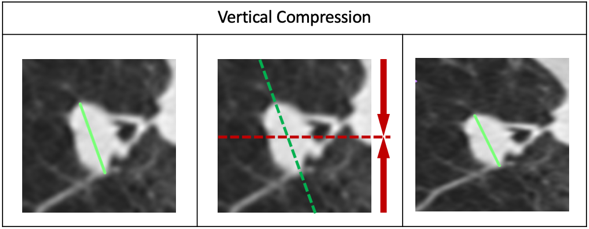

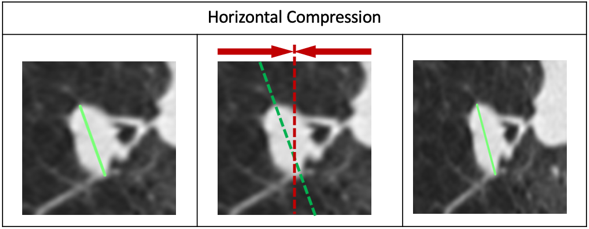


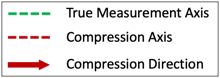


**Supplemental Figure 1.** Example of effect of different compression axis on measurement value.

As an example, when true measurement axis approximates to vertical axis, the numerical value of the longest measurement does not change significantly when lesion image is compressed horizontally using vertical compression axis, Supplemental Figure 1. On the contrary, the numerical value of the longest measurement decreases significantly when vertical compression (i.e. horizontal compression axis) is used due to the discrepancy between the true measurement axis and compression axis.

Images containing target lesion were rotated from 0 to 360 degrees with increment of 3 degree. The vertical compression was performed on the rotated images to find images which yield the maximum numerical value of measurement after the compression, Supplemental Figure 2.

**Supplemental Figure 2.** Compression at various angles and the corresponding measurement. The images were rotated, horizontally compressed, and measured to identify compression axes minimally affecting the numerical value of measurement.

Using scatter-plot of compression axis and the corresponding measurement value, a pair of images apart from each other by approximately 180 degrees and yielding the largest numerical value of measurement were identified. Since their compression axis had a minimal effect on the numerical value of measurement, the axis was selected as the longest measurement axis for the visualization.

**- Measurement Vertex**

The measurement vertex was acquired using some basic masking and vector shape building techniques.

**Supplemental Figure 3.** Use of masking concave hull to identify start point of measurement. Masking based on Canny edge detector was performed around the arbitrary input point inside the target lesion. A point yielding the longest measurement for the given fixed axis was selected as the start point of measurement.

First, the Canny edge detector was used to segmentize the anatomic structures adjacent to the given arbitrary point locating inside the target lesion, Supplemental Figure 3. The segmentation resulted in a set of boundaries including both lesion and non-lesion areas in the field of view. Given the axis of the longest measurement, a pair of points which locate on the boundary and yield the longest possible measurement crossing the segmentized area was selected. A single point locating close to the center of mass based the segmentation was selected as a vertex of the longest measurement for the visualization.
